# Supplementary material for: Whole-Genome Sequencing of Three Native Cattle Breeds Originating From the Northernmost Cattle Farming Regions
Source: Front Genet. 2019 Jan 11;9:728. doi: 10.3389/fgene.2018.00728 (PMC6336893; doi:10.3389/fgene.2018.00728)
Supplement: Supplementary file 1 [file Table_1.DOCX]

Supplementary Material

Whole-genome sequencing of three native cattle breeds originating from the northernmost cattle farming regions

Melak Weldenegodguad, Ruslan Popov, Kisun Pokharel, Innokentyi Ammosov, Ming Yao, Zoya Ivanova and Juha Kantanen*

*** Correspondence:** Corresponding Author: Juha.kantanen@luke.fi

# Supplementary Data

**Supplementary Data 1:** Genes containing frameshift indels in Eastern Finncattle (Supplementary_Data_1.xlsx).

**Supplementary Data 2:** Genes containing frameshift indels in Western Finncattle (Supplementary_Data_2.xlsx).

**Supplementary Data 3:** Genes containing frameshift indels in Yakutian Finncattle (Supplementary_Data_3.xlsx).

**Supplementary Data 4:** Genes containing nsSNPs > 5 in Eastern Finncattle (Supplementary_Data_4.xlsx).

**Supplementary Data 5:** Genes containing nsSNPs > 5 in Western Finncattle (Supplementary_Data_5.xlsx).

**Supplementary Data 6:** Genes containing nsSNPs > 5 in Yakutian Finncattle (Supplementary_Data_6.xlsx).

**Supplementary Data 7:** GOES enrichment result for the genes containing nsSNPs > 5 in Eastern Finncattle **(**Supplementary_Data_7.xlsx**).**

**Supplementary Data 8:** GOES enrichment result for the genes containing nsSNPs > 5 in Western Finncattle **(**Supplementary_Data_8.xlsx**)**

**Supplementary Data 9:** GO enrichment results for the genes containing nsSNPs > 5 in Yakutian Finncattle **(**Supplementary_Data_9.xlsx**).**

**Supplementary Data 10:** GO enrichment results for the genes containing frameshift in Eastern Finncattle (Supplementary_Data_10.xlsx).

**Supplementary Data 11:** GO enrichment results for the genes containing frameshift in Western Finncattle (Supplementary_Data_11.xlsx).

**Supplementary Data 12:** GO enrichment results for the genes containing frameshift in Yakutian Finncattle (Supplementary_Data_12.xlsx).

**Supplementary Data 13:** Candidate genes with high signature of selection signal identified by SweeD analysis in Eastern Finncattle (Supplementary_Data_13.xlsx).

**Supplementary Data 14:** Candidate genes with high signature of selection signal identified by SweeD analysis in Western Finncattle (Supplementary_Data_14.xlsx).

**Supplementary Data 15:** Candidate genes with high signature of selection signal identified by SweeD analysis in Yakutian Finncattle (Supplementary_Data_15.xlsx).

**Supplementary Data 16:** Selective sweep genes containing nsSNPs > 5 in Eastern Finncattle (Supplementary_Data_16.xlsx).

**Supplementary Data 17:** Selective sweep genes containing nsSNPs > 5 in Western Finncattle (Supplementary_Data_17.xlsx).

**Supplementary Data 18:** Selective sweep genes containing nsSNPs > 5 in YaKutian Finncattle (Supplementary_Data_18.xlsx).

**Supplementary Data 19:** GO enrichment result for the genes showing high signature of selection signal identified by SweeD analysis in Eastern Finncattle (Supplementary_Data_19.xlsx).

**Supplementary Data 20:** GO enrichment result for the genes showing high signature of selection signal identified by SweeD analysis in Western Finncattle (Supplementary_Data_20.xlsx).

**Supplementary Data 21:** GO enrichment result for the genes showing high signature of selection signal identified by SweeD analysis in Yakutian Finncattle (Supplementary_Data_21.xlsx).

# Supplementary Figures and Tables

## Supplementary Figures

#
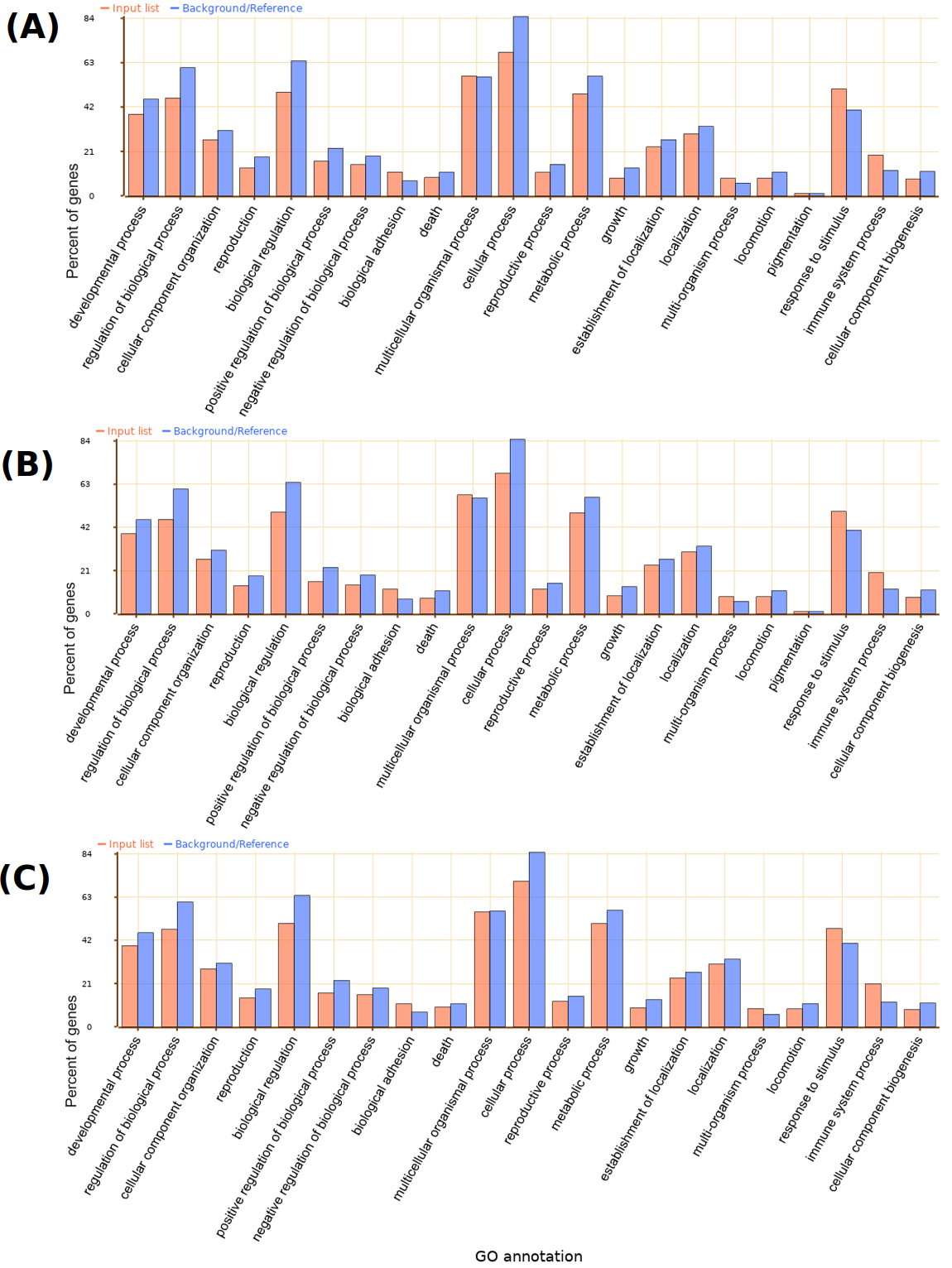


### Figure S1. GO annotation of the genes harboring highest number of nsSNPS in Eastern Finncattle (A) Western Finncattle (B) and Yakutian cattle (C). The x-axis indicates the GO terms and the Y-axisis indicates the percentage of the query genes in different GO terms. The blue bar shows the percentage of genes among the query genes (nsSNP-containing genes), and the green bar shows the percentage of genes present in background (Bovine Ensembl gene).


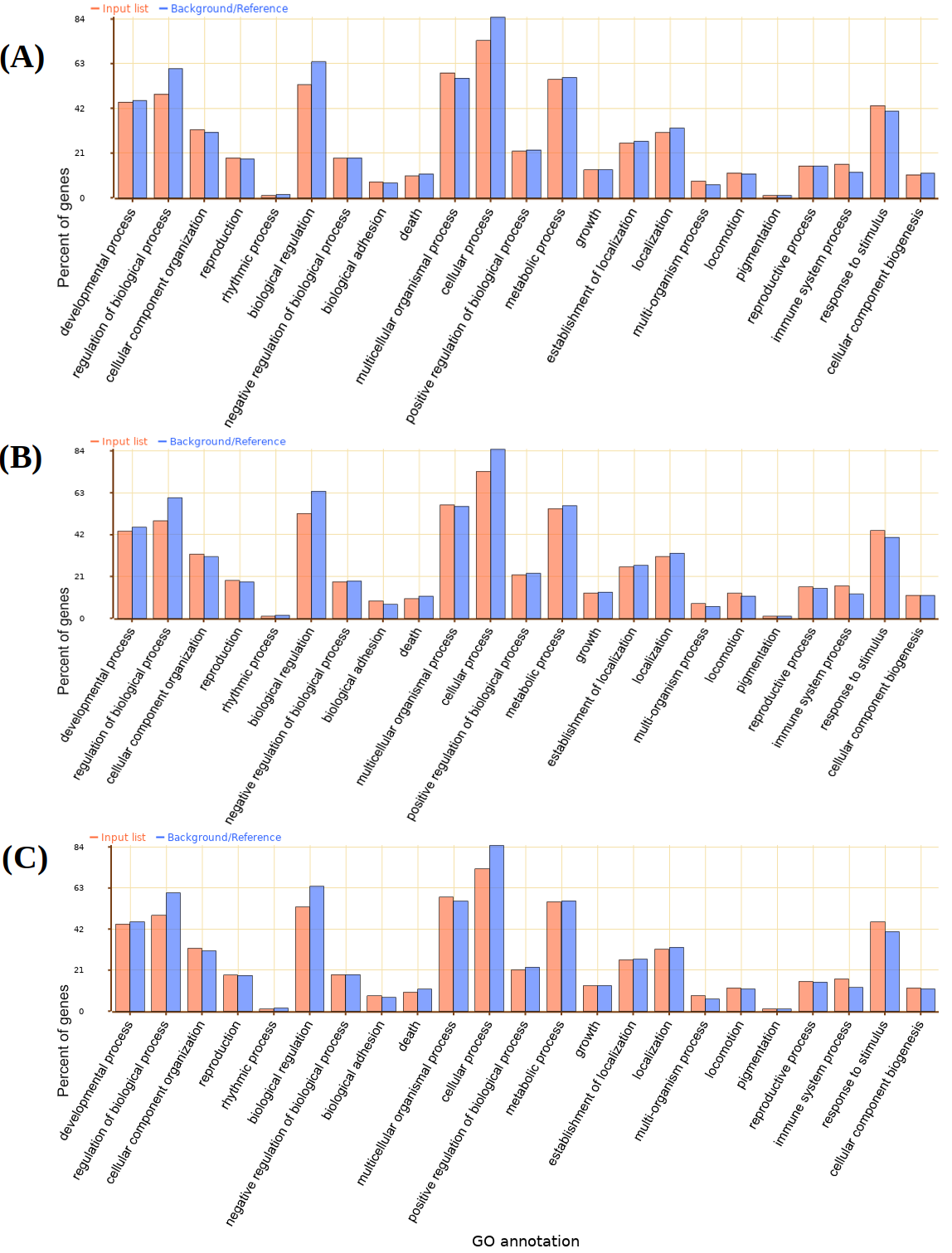


### Figure S2. GO annotation of the genes harboring frameshift indels in Eastern Finncattle (A) Western Finncattle (B) and Yakutian cattle (C). The x-axis indicates the GO terms and the Y-axis indicates the percentage of the query genes in different GO terms. The blue bar shows the percentage of genes among the query genes (frameshift-containing genes), and the green bar shows the percentage of genes present in background (Bovine Ensembl annotation).

**
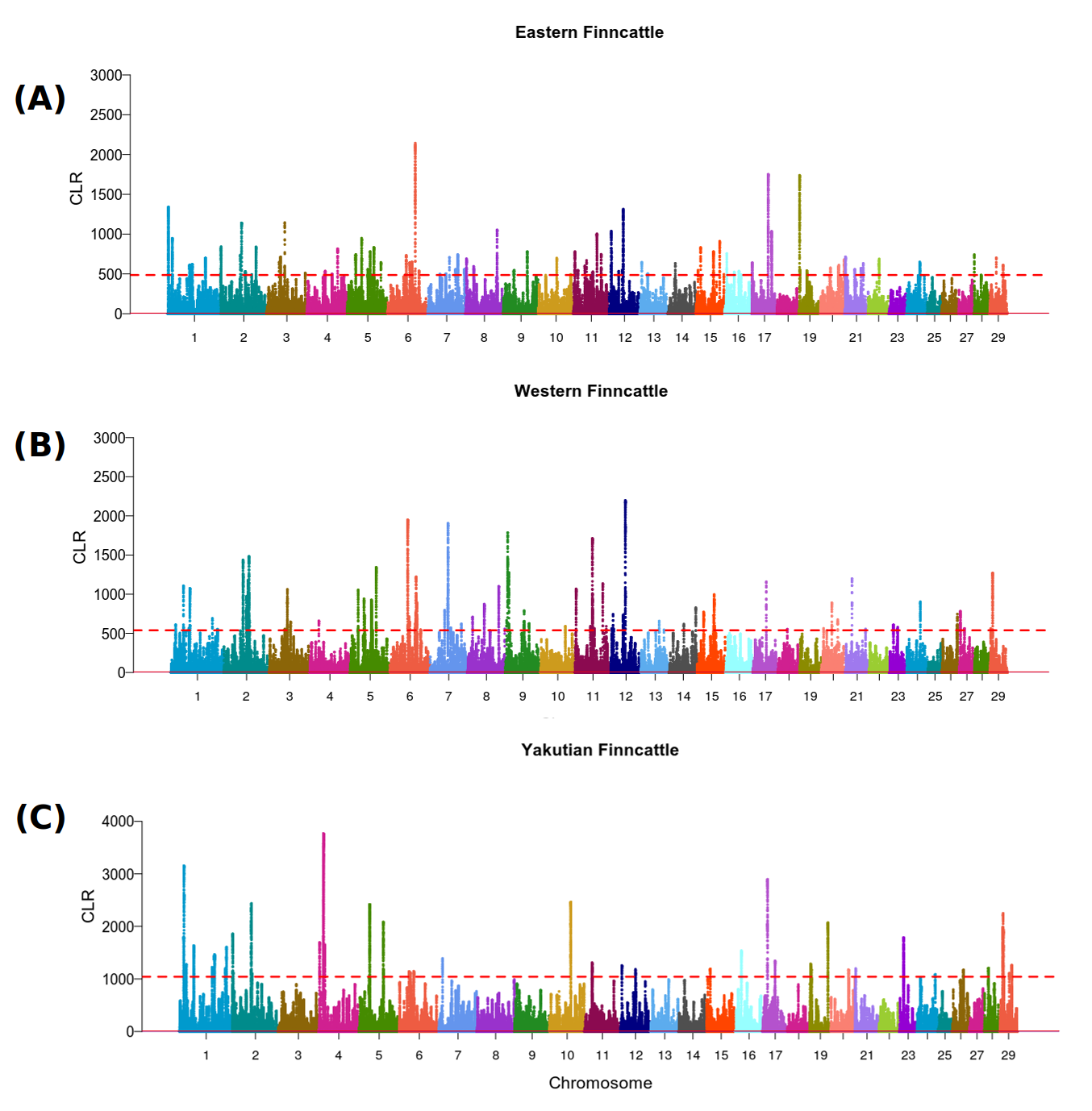
**

**Figure S3**. Manhattan plots of selective sweep of the Northern-Eursian breed **(A)** Eastern Finncattle **(B)** Western Fincattle and **(C)** Yakutian cattle


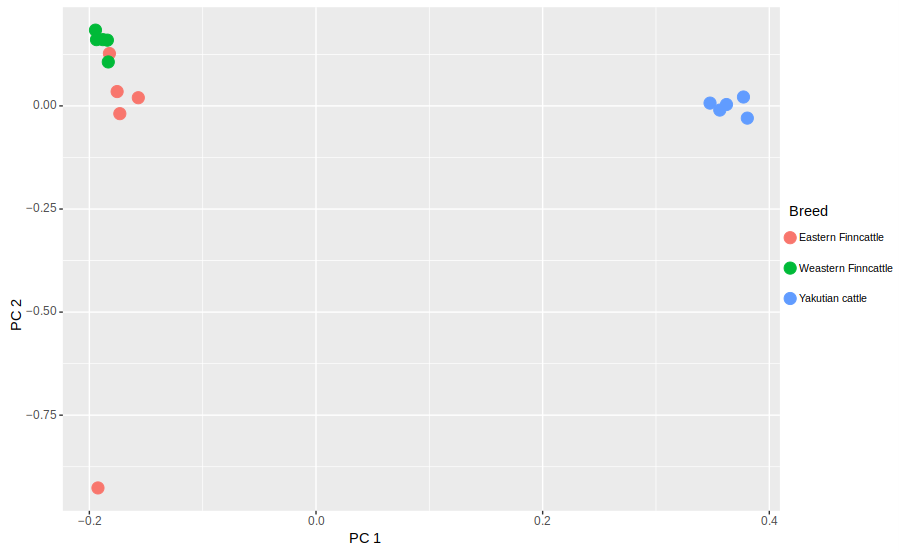


### Figure S4. PCA. Principal component analysis of 15 Northern-Eurasian cattle samples. Eastern Finncattle genomes are indicated by red, Western Finncatle by green, Yakutian cattle by blue.


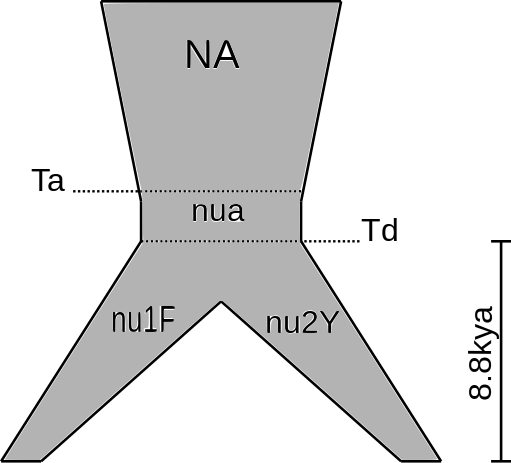


**Figure S5.** Dadi model. Inferred demographic history for the Finncattle and Yakutian cattle. Time depth parameters are: Ta, referees to the ancestral population size change; Td the divergence time between Finncatle and Yakutian cattle.

## Supplementary Tables

**Table S1.** Whole genome sequencing and mapping statistics of Eastern Finncattle, Western Finncattle and Yakutian cattle.

| Breed | Sample name | Accession | PE length (bp) | Raw reads (M) | Clear raw base (GB) | Mapped reads (M) | Mapped base (GB) | Uniq map reads | read mapping rate (%) | Base mapping rate (%) | Depth (x) |
| --- | --- | --- | --- | --- | --- | --- | --- | --- | --- | --- | --- |
| Eastern Finncattle | sample_1 | ERS2647064 | 100 | 351.9 | 35.19 | 347.8 | 34.78 | 314.1 | 98.84 | 98.51 | 13.18 |
|  | sample_2 | ERS2647065 | 100 | 348.5 | 34.85 | 344.23 | 34.42 | 314.21 | 98.78 | 98.39 | 13.05 |
|  | sample_3 | ERS2647066 | 100 | 345.7 | 34.57 | 341.72 | 34.17 | 311.37 | 98.86 | 98.29 | 12.94 |
|  | sample_4 | ERS2647067 | 100 | 359.1 | 35.91 | 354.59 | 35.46 | 322.53 | 98.74 | 98.46 | 13.45 |
|  | sample_5 | ERS2647068 | 100 | 358.5 | 35.85 | 353.76 | 35.38 | 322.23 | 98.66 | 98.43 | 13.42 |
| Western Finncattle | sample_6 | ERS2647069 | 100 | 336.8 | 33.68 | 323.88 | 32.39 | 298.22 | 96.17 | 98.42 | 12.61 |
|  | sample_7 | ERS2647070 | 100 | 354.2 | 35.42 | 350.03 | 35 | 322.52 | 98.81 | 98.16 | 13.27 |
|  | sample_8 | ERS2647071 | 100 | 353.4 | 35.34 | 348.73 | 34.87 | 318.45 | 98.68 | 98.22 | 13.23 |
|  | sample_9 | ERS2647072 | 100 | 343.9 | 34.39 | 336.3 | 33.63 | 309.93 | 97.78 | 98.2 | 12.88 |
|  | sample_10 | ERS2647073 | 100 | 347.3 | 34.73 | 341.84 | 34.18 | 313.77 | 98.42 | 98.12 | 13.01 |
| Yakutian cattle | sample_11 | ERS2647074 | 100 | 357.7 | 35.77 | 352.2 | 35.22 | 318.53 | 98.46 | 98.68 | 13.4 |
|  | sample_12 | ERS2647075 | 100 | 341.9 | 34.19 | 337.37 | 33.74 | 309.48 | 98.67 | 98.47 | 12.8 |
|  | sample_13 | ERS2647076 | 100 | 336,7 | 33.67 | 332.23 | 33.22 | 308.32 | 98.67 | 98.42 | 12.61 |
|  | sample_14 | ERS2647077 | 100 | 343.3 | 34.33 | 338.27 | 33.83 | 309.84 | 98.54 | 98.4 | 12.86 |
|  | sample_15 | ERS2647078 | 100 | 332 | 33.2 | 327.4 | 32.74 | 299.75 | 98.61 | 98.35 | 12.43 |
| Total |  |  |  |  | 521.09 |  |  |  |  |  |  |

**Table S2.** Summary of SNP Calling

|  | Eastern Finncattle | Western Finncattle | Yakutian cattle | Overall sample |
| --- | --- | --- | --- | --- |
| Number of SNPs | 11,017,215 | 10,543,290 | 12,242,166 | 17,454757 |
| Number of Indels | 1,271,528 | 1,188,892 | 1,374,577 | 2,121,016 |
| Ts/Tv ratio | 2.20 | 2.20 | 2.23 |  |
| Average number of SNPs per individual | 5,729,403 | 6,028,950 | 7,122,285 |  |
| Homozygous to heterozygous ratio | 1:1.03 | 1:1.58 | 1:1.48 |  |
| Proportion of homozygotic SNP-loci | 49.50% | 38.72% | 40.20% |  |

**Table S3** Summary of identified SNP for individual samples

| **Breed** | **Sample name** | **Total SNP** | **Heterozygous** | **Homozygous** |
| --- | --- | --- | --- | --- |
| **Eastern Finncattle** | sample_1 | 6234818 | 3999913 | 2248180 |
|  | sample_2 | 5705391 | 2851167 | 2865738 |
|  | sample_3 | 5111001 | 1666010 | 3454670 |
|  | sample_4 | 6005833 | 3490602 | 2527918 |
|  | sample_5 | 5589970 | 2665175 | 2936076 |
| **Western Finncattle** | sample_6 | 6038990 | 3773669 | 2277053 |
|  | sample_7 | 5942778 | 3517945 | 2436501 |
|  | sample_8 | 6142242 | 3906248 | 2248525 |
|  | sample_9 | 6003996 | 3635873 | 2379742 |
|  | sample_10 | 6016745 | 3643076 | 2385307 |
| **Yakutian cattle** | sample_11 | 7121633 | 4189795 | 2948384 |
|  | sample_12 | 7111794 | 4277914 | 2850715 |
|  | sample_13 | 7161700 | 4352954 | 2825739 |
|  | sample_14 | 7195522 | 4376069 | 2836062 |
|  | sample_15 | 7020777 | 4101255 | 2935739 |

**Table S4** Summary statistics of the functional class of the identified SNP for each individual in the respective breed

| **Breed** | **Sample name** | **Total SNP** | **Intergenic** | **Intronic** | **Total in Exonic** | **Non-synonymous** | **Stopgain** | **Stoploss** | **Synonymous** | **gene containg nsSNPs** |
| --- | --- | --- | --- | --- | --- | --- | --- | --- | --- | --- |
| **Eastern Finncattle** | sample_1 | 6234818 | 4530110 | 1556344 | 41275 | 17564 | 151 | 15 | 23541 | 7235 |
|  | sample_2 | 5705391 | 4149454 | 1420252 | 37907 | 16086 | 123 | 15 | 21677 | 6862 |
|  | sample_3 | 5111001 | 3705981 | 1282566 | 34402 | 14694 | 135 | 14 | 19558 | 6403 |
|  | sample_4 | 6005833 | 4373732 | 1491060 | 39045 | 16556 | 135 | 14 | 22336 | 6951 |
|  | sample_5 | 5589970 | 4062359 | 1394518 | 36749 | 15767 | 128 | 13 | 20837 | 6601 |
| **Western Finncattle** | sample_6 | 6038990 | 4398754 | 1500984 | 38397 | 16337 | 137 | 13 | 21904 | 6946 |
|  | sample_7 | 5942778 | 4325591 | 1482038 | 30047 | 15805 | 136 | 12 | 21086 | 6638 |
|  | sample_8 | 6142242 | 4474577 | 1526479 | 38809 | 16463 | 128 | 14 | 22200 | 6919 |
|  | sample_9 | 6003996 | 4359545 | 1506809 | 38241 | 16295 | 138 | 12 | 21794 | 6816 |
|  | sample_10 | 6016745 | 4370743 | 1506865 | 38353 | 16342 | 144 | 10 | 21855 | 6889 |
| **Yakutian cattle** | sample_11 | 7121633 | 5145913 | 1805094 | 47332 | 19529 | 174 | 13 | 27610 | 7667 |
|  | sample_12 | 7111794 | 5126803 | 1815662 | 47086 | 19534 | 173 | 14 | 27361 | 7610 |
|  | sample_13 | 7161700 | 5175545 | 1816302 | 47506 | 19699 | 178 | 14 | 27609 | 7638 |
|  | sample_14 | 7195522 | 5202507 | 1823429 | 46875 | 19289 | 161 | 11 | 27409 | 7618 |
|  | sample_15 | 7020777 | 5072723 | 1781955 | 46318 | 19280 | 167 | 16 | 26850 | 7639 |

**Table S5** Statistics of the functional class of SNPs found in selective sweep

| Consequences (all) | Eastern Finncattle | Weastern Finncattle | Yakutian cattle |
| --- | --- | --- | --- |
| splice_donor_variant | 9 | 21 | 10 |
| splice_acceptor_variant | 2 | 6 | 4 |
| stop_gained | 3 | 4 | 5 |
| stop_lost | 1 | 0 | 1 |
| start_lost | 5 | 1 | 3 |
| missense_variant | 670 | 755 | 577 |
| splice_region_variant | 103 | 113 | 135 |
| synonymous_variant | 741 | 864 | 690 |
| stop_retained_variant | 1 | 1 | 1 |
| coding_sequence_variant | 2 | 5 | 3 |
| 5_prime_UTR_variant | 77 | 105 | 68 |
| 3_prime_UTR_variant | 274 | 346 | 227 |
| non_coding_transcript_exon_variant | 35 | 59 | 48 |
| intron_variant | 42075 | 50431 | 44013 |
| non_coding_transcript_variant | 1 | 59 | 49 |
| upstream_gene_variant | 5817 | 8350 | 7129 |
| downstream_gene_variant | 5259 | 8012 | 6740 |
| intergenic_variant | 148795 | 134527 | 132227 |

**Table S6.** Candidate selective sweep genes not annotated and conting nsSNPs > 5

| **Eastern Finncattle** | **Western Finncattle** | **Yakutian cattle** |
| --- | --- | --- |
| ENSBTAG00000026133 | ENSBTAG00000012201 | ENSBTAG00000015464 |
| ENSBTAG00000027899 | ENSBTAG00000012326 | ENSBTAG00000025621 |
| ENSBTAG00000039755 | ENSBTAG00000018399 | ENSBTAG00000031082 |
| ENSBTAG00000040257 | ENSBTAG00000025621 | ENSBTAG00000035333 |
| ENSBTAG00000040473 | ENSBTAG00000045932 | ENSBTAG00000035701 |
| ENSBTAG00000045571 |  | ENSBTAG00000037490 |
|  |  | ENSBTAG00000039016 |
|  |  | ENSBTAG00000048275 |

**Table S7**. Dadi inferred parameters

| **Parameters** | **Fitted** | **CI** |
| --- | --- | --- |
| NA | 43,116 |  |
| nua | 51,883 | 52,108 – 51,658 |
| nu1F | 5,487 | 5,516 – 5,458 |
| nu2Y | 5,920 | 5,924 – 5,914 |
| Ta | 411,715 | 413,965 - 409,465 |
| Td | 8,822 | 8,869 – 8,775 |
| Maximum liklihood | -43,913.337422 |  |
